# Telomere to Telomere Genome Assembly and Efficient Transformation and Genome Editing in *Populus euphratica*

**DOI:** 10.1111/pbi.70609

**Published:** 2026-03-14

**Authors:** Yi An, Rui Yang, Song Yang, Xiaohui Gou, Yuhui Li, Yan Dong, Yangyan Zhou, Xiao Han

**Affiliations:** ^1^ National Key Laboratory for Development and Utilization of Forest Food Resources, Zhejiang Key Laboratory of Forest Genetics and Breeding, Plant Cell Wall Research Centre, College of Forestry and Biotechnology Zhejiang A&F University Hangzhou China; ^2^ China Flower Association Beijing China; ^3^ Shandong Key Laboratory of Fruit and Forest Germplasm Resources Innovation and Precision Breeding, Salver Academy of Botany Rizhao Shandong China

**Keywords:** gap‐free genome assembly, genome editing, *Populus euphratica*, transgenics

*Populus euphratica* Oliv. is a key desert tree, blocking sand, stabilizing dunes, ameliorating microclimates, and providing wildlife habitats, vital for ecological stability. In addition, its outstanding drought, cold, and salinity tolerance, and features like heterophylly, make it valuable for studying plant adaptation and growth. However, incompatibility of hybridization, poor rooting, and limited genetic research platforms hinder conservation and utilisation. Consequently, the development of robust biotechnology platforms is urgently needed in *P. euphratica*.

Complete genomic information facilitates the elucidation of gene function and the efficient application of genome editing, but the available genome assemblies of *P. euphratica* remain discontinuous and incomplete due to gaps and missing telomeres (Zhang et al. 2022, 2020). Here, we assembled the telomere‐to‐telomere (T2T) haplotype‐resolved genome of *P. euphratica* ‘ZH1’ (Figure 1A, Table S1, and Data S1). The gapless T2T genome, composed of 38 chromosomes and 76 telomeres, encompassed two haploids of 506.85 and 501.23 Mb (Figures S1 and S2, Table S2). The contig N50 values of two haploids were 24.99 and 24.72 Mb, respectively, and the BUSCO assessment showed 98.9% completeness, significantly improving the continuity and completeness (Table S3). The mapping rates for HiFi, ONT, and NGS reads were 99.92%, 99.35%, and 98.94%, respectively. The genome continuity inspector (GCI) assessments for two haploids were 93.07 and 96.26, respectively. The consensus quality value (QV) assessments were 72.33 and 73.41, respectively, higher than other T2T assemblies of *Populus* species (Table S4). The repeat element was 58.37% and 58% of the genome assembly (Table S5). The rDNAs were especially enriched on Chr9 (Tables S6 and S7). A total of 38 257 and 38 176 protein‐coding genes were identified, 98.56% of which were confirmed entirely using 113 RNA‐seq samples, and 97.18% were annotated by public databases (Tables S8 and S9). Comparative genomic analysis showed that the T2T assembly filled gaps in the previous genome and annotated novel genes (Figure S3). These results suggest the availability of a complete genome assembly of *P. euphratica*.

**FIGURE 1:**
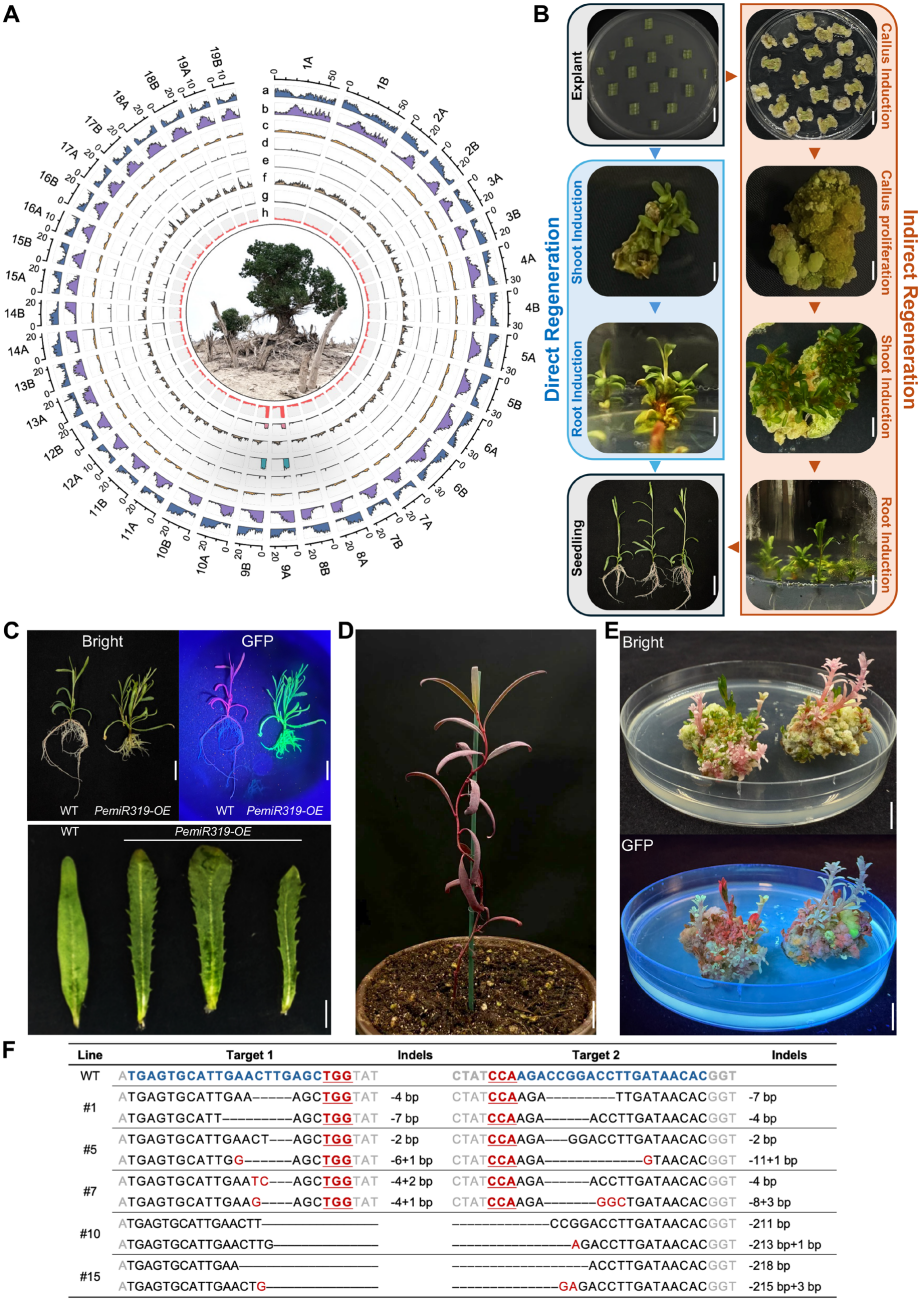
A Telomere‐to‐Telomere genome assembly and genome editing of *Populus euphratica*. (A) The genome assembly of *P. euphratica* ‘ZH1’. (a) gene density; (b) LTR density; (c) DNA transposon density; (d) LINE density; (e) SINE density; (f) helitron density; (g) TR density; (h) GC content. The central figure shows *Populus euphratica* in Xinjiang, China. (B) Indirect and direct regeneration processes of *P. euphratica*. The indirect process: The callus is induced from the explants and then regenerates into a shoot. The direct process: The shoot is induced directly from the explants. (C) Variation of the leaf margin of *PemiR319*‐overexpressing *P. euphratica*. A green fluorescence signal is observed in the transgenic *P. euphratica*. (D) One‐month‐old RUBY‐overexpressing *P. euphratica*. (E) Knockout of the *PDS* gene in *P. euphratica* by CRISPR/Cas9. The pale or pink shoots are the positive genome‐edited *P. euphratica*. (F) The editing events of PePDS‐KO lines. The sequences in bold are target sites. Bar = 1 cm.

Despite the well‐established transformation systems for *Populus*, their application is currently limited to only a few species and cultivars. Notably, the regeneration and transformation systems for *P. euphratica* have remained elusive, significantly hindering functional characterisation of genes and the genetic application. Here, we developed indirect and direct strategies for regenerating *P. euphratica*. In an indirect way, sterilised leaves and petioles were transferred into callus‐induction media to initiate callus formation. The healthy callus was transferred to shoot‐induction media to initiate adventitious buds and elongated shoots. The elongated shoots were transferred to root‐induction media to form complete plants. In a direct manner, sterilised explants were to initiate buds directly (Figure 1B). Both indirect and direct processes exhibited high regeneration efficiency and could be used for transgenic applications of *P. euphratica* (Table S10 and Data S2).

The variation of leaf shape constitutes one of the distinctive characteristics of *P. euphratica* in response to environmental change. MiR319 plays a significant role in regulating leaf morphology (Cheng et al. 2021). We cloned the *PemiR319* gene from *P. euphratica*, constructed an overexpressing vector pXHKFG‐PemiR319, and transformed it into *P. euphratica* (Data S3 and File S1). The green fluorescence signal was observed across the transgenic *P. euphratica* (Figure 1C). The transgenic *P. euphratica* exhibited serrated leaves (Figure 1D), a phenotype consistent with *PagmiR319*‐overexpressing 84 K poplar (Figure S4).

The RUBY reporter has been widely used as a marker in plant transgenics. The RUBY reaction necessitates sequential catalysis by three enzymes. Thus, we constructed a multiple‐gene coexpression system pXHKFG‐RUBY (Data S3). Transgenic callus and shoots with RUBY exhibited a distinct red colour, markedly differentiated from its negative counterparts (Figure S5), and the rooting *P. euphratica* plants also exhibited red (Figure 1E).

Efficient gene editing requires precise selection of editing targets, which necessitates reliance on high‐quality genome information of the recipient. Based on the CRISPR/Cas9 principle, we employed CRISPR‐Local (Sun et al. 2019) for genome‐wide target selection by integrating the T2T genome of *P. euphratica*, thereby enhancing the specificity of target sites across the genome and reducing off‐target events. We selected two target sites of the phytoene desaturase (PDS) gene in *P. euphratica* (*PePDS*) and constructed a two‐site editing system pXHGCK‐PePDS (Data S3 and File S2). The positive *PePDS*‐edited pink shoots were successfully achieved (Figure 1F). The sequencing results showed that different editing events had occurred at both target sites of the *PePDS* gene (Figure 1G), whereas the potential top‐off‐target sites had no edits (Figure S5). The editing efficiency of the direct regeneration way was lower than that of the indirect way. In the direct way, there were fewer homozygous mutations, and most were chimeric or unmodified. In the indirect way, biallelic and homozygous edits were predominant (Table S11). To our knowledge, this was the first report of genome editing in *P. euphratica*.

In summary, this work first provides a high‐quality T2T gap‐free genome assembly of *P. euphratica* (ZH1‐T2T), serving as a critical foundation for gene exploration and enabling efficient target selection for genome editing. Secondly, we have developed an efficient regeneration system for *P. euphratica*, providing an essential method for propagating and proliferating superior clonal lines. Thirdly, we have established an efficient transformation system for *P. euphratica*, offering significant support for gene characterisation and genetic improvement. We have also successfully conducted genome editing in *P. euphratica*. These studies collectively provide robust technical support for the conservation and utilisation of *P. euphratica*.

## Author Contributions

X.H. supervised the project. X.H. and Y.A. wrote the paper and performed the regeneration. R.Y. and S.Y. performed the transgenics. R.Y. and X.G. cultivated the samples. X.H. and Y.L. performed genome assembly. Y.D. and Y.Z. collected samples.

## Funding

This work was supported by the Science and Technology Innovation 2030‐Major Project (2023ZD0405702‐05) and the National Natural Science Foundation of China (32371902).

## Conflicts of Interest

The authors declare no conflicts of interest.

## Supporting information


**Figures S1–S6**, **Tables S1–S11** and **Data S1–S3**.

## Data Availability

All raw sequencing data and the genome assembly have been deposited in the CNSA under accession number CNP0008260.
