# Supplementary material for: Telomere to Telomere Genome Assembly and Efficient Transformation and Genome Editing in Populus euphratica
Source: Plant Biotechnol J. 2026 Mar 14;24(6):4282–4. doi: 10.1111/pbi.70609 (PMC13205687; doi:10.1111/pbi.70609)
Supplement: Supplementary file 1 — Figures S1–S6, Tables S1–S11 and Data S1–S3. [file PBI-24-4282-s001.pdf]

Supplemental materials

Supplemental Tables 1-11

Supplemental Figures 1-6

Supplemental Notes 1-3

Supplemental Table 1. Sequencing statistics of the *Populus euphratica* genome.

Statistic	Sequencing
PacBio HiFi data (bp)	57,811,969,995
Sequencing depth (×)	> 110
Reads number	3,240,433
Average read length (bp)	17,840.8
Reads N50 (bp)	17,816
GC content (%)	32.5
ONT ultra-long data (bp)	51,603,108,244
Sequencing depth (×)	> 100
Reads number	1,528,394
Average read length (bp)	33,763
Reads N50 (bp)	66,411
GC content (%)	32.88
Hi-C data (bp)	67,532,739,609
Sequencing depth (×)	> 130
BGI short-read data (bp)	57,318,846,498
Sequencing depth (×)	> 100

Supplemental Table 2. Statistics of the chromosome length and telomere positions in *Populus euphratica*.

ID	Length (bp)	Gap number	Upstream number	Downstream number	Upstream position range	Downstream position range
**chr1A**	58,655,440	0	2,722	2,575	8-19,861	58,636,986-58,655,434
**chr1B**	57,436,625	0	2,808	2,810	9-20,000	57,416,627-57,436,625
**chr2A**	29,190,506	0	2,806	2,805	5-19,996	29,170,507-29,190,500
**chr2B**	29,226,842	0	2,486	2,808	5-18,693	29,206,848-29,226,838
**chr3A**	25,259,859	0	2,808	2,808	8-19,999	25,239,860-25,259,852
**chr3B**	25,416,724	0	2,806	2,804	8-19,995	25,396,726-25,416,724
**chr4A**	33,064,021	0	2,741	2,809	9-19,999	33,044,025-33,064,020
**chr4B**	31,883,822	0	2,809	2,807	3-19,994	31,863,824-31,883,818
**chr5A**	32,160,037	0	2,808	2,809	8-19,999	32,140,041-32,160,032
**chr5B**	32,219,412	0	2,808	2,806	9-20,000	32,199,418-32,219,407
**chr6A**	36,272,084	0	2,777	2,805	9-19,969	36,252,090-36,272,071
**chr6B**	35,937,965	0	2,133	2,809	2-16,168	35,917,970-35,937,961
**chr7A**	21,902,343	0	2,805	947	31-19,994	21,884,570-21,902,334
**chr7B**	21,888,210	0	2,808	2,806	2-20,000	21,868,216-21,888,208
**chr8A**	24,985,422	0	2,396	2,806	10-17,810	24,965,423-24,985,421
**chr8B**	24,722,656	0	2,809	2,809	4-19,995	24,702,658-24,722,656
**chr9A**	24,236,992	0	2,805	2,805	29-19,999	24,216,996-24,236,990
**chr9B**	24,111,751	0	2,808	2,694	2-19,995	24,091,755-24,111,710
**chr10A**	27,843,494	0	2,808	2,809	5-19,997	27,823,495-27,843,493
**chr10B**	26,857,932	0	2,808	2,299	8-19,999	26,841,547-26,857,901
**chr11A**	22,869,178	0	2,805	2,783	7-19,998	22,849,364-22,869,172
**chr11B**	23,179,793	0	2,803	2,808	31-19,994	23,159,798-23,179,789
**chr12A**	19,679,398	0	2,804	2,501	5-19,994	19,660,680-19,679,391
**chr12B**	20,325,021	0	2,808	2,804	10-19,994	20,305,022-20,325,014
**chr13A**	22,974,091	0	2,804	2,808	32-19,995	22,954,092-22,974,090
**chr13B**	22,315,074	0	2,781	2,806	6-19,995	22,295,080-22,315,072
**chr14A**	23,009,388	0	2,809	2,809	2-19,994	22,989,392-23,009,383
**chr14B**	22,403,171	0	2,660	2,749	1-19,080	22,383,176-22,403,167
**chr15A**	21,199,491	0	2,804	2,807	14-19,997	21,179,497-21,199,489
**chr15B**	20,497,641	0	2,808	2,808	10-19,994	20,477,643-20,497,641
**chr16A**	19,646,428	0	2,807	2,808	11-20,000	19,626,435-19,646,428
**chr16B**	20,425,367	0	2,805	2,808	6-19,998	20,405,373-20,425,357
**chr17A**	20,869,992	0	2,806	2,809	24-19,994	20,849,999-20,869,992
**chr17B**	20,621,266	0	2,808	2,749	5-19,996	20,601,273-20,621,266
**chr18A**	23,212,058	0	2,808	2,809	10-19,994	23,192,059-23,212,050
**chr18B**	22,556,843	0	2,807	2,122	6-20,000	22,541,292-22,556,836
**chr19A**	19,817,444	0	2,731	2,542	202-19,988	19,799,092-19,817,440
**chr19B**	19,202,853	0	2,258	2,809	8-16,709	19,182,856-19,202,847

Supplemental Table 3. Comparison of statistics of the published *Populus euphratica* genomes.

	Ma et al., 2013	Zhang et al., 2020	Zhang et al., 2022		ZH1-T2T	
			Female	Male	Hap A	Hap B
**Assembly method**	Illumina + Fosmid	Illumina + PacBio + Hi-C	Illumina + PacBio + Hi-C		HiFi + ONT + Hi-C	
**Assembly level**	Contig	Chromosome	Chromosome		T2T	
**Assembly size (bp)**	496,523,805	574,348,232	511,252,577	1,032,482,259	506,847,666	501,228,968
**GC content (%)**	32.33	32.38	32.29	32.23	32.71	32.67
**Chromosome number**	—	19	19	38	19	19
**Heterozygosity**	~0.5%	~0.52%	0.86%	1.99%	1.33~1.37%	
**Contig number**	32,882	1,573	432	3,347	19	19
**N50 (bp)**	40,438	900,000	2,039,164	892,045	24,985,422	24,722,656
**Gap number**	—	—	—	—	0	0
**Telomere number**	—	—	—	—	38	38
**Genome BUSCO (%)**	94.35	95.6	92.5	87.9	98.9	98.9
**TE content (%)**	40.52	56.95	64.41	52.38	58.37	58
**Number of protein-coding genes**	34,279	36,606	36,792	70,370	38,257	38,176
**Average length of protein-coding gene (bp)**	3,442.54	3,454.27	3,231.15	3,242.12	3,047.63	3,023.21
**Average length of CDS (bp)**	1,272.35	1,132.89	1,082.36	1,102.75	1,161.59	1,162.07
**Average number of exons per transcript**	5.19	5.52	4.93	5.00	4.92	4.92
**Gene BUSCO (%)**	—	91.1%	86.6	84.3	98.5	98.3

References

Ma, T., Wang, J., Zhou, G., Yue, Z., Hu, Q., Chen, Y., Liu, B., Qiu, Q., Wang, Z., Zhang, J., Wang, K., Jiang, D., Gou, C., Yu, L., Zhan, D., Zhou, R., Luo, W., Ma, H., Yang, Y., Pan, S., Fang, D., Luo, Y., Wang, X., Wang, G., Wang, J., Wang, Q., Lu, X., Chen, Z., Liu, J., Lu, Y., Yin, Y., Yang, H., Abbott, R.J., Wu, Y., Wan, D., Li, J., Yin, T., Lascoux, M., Difazio, S.P., Tuskan, G.A., Wang, J. and Liu, J. (2013) Genomic insights into salt adaptation in a desert poplar. *Nature communications* **4**, 2797.

Zhang, Z., Chen, Y., Zhang, J., Ma, X., Li, Y., Li, M., Wang, D., Kang, M., Wu, H., Yang, Y., Olson, M.S., DiFazio, S.P., Wan, D., Liu, J. and Ma, T. (2020) Improved genome assembly provides new insights into genome evolution in a desert poplar (Populus euphratica). *Mol Ecol Resour* **20**, 781-794.

Zhang, S., Wu, Z., Ma, D., Zhai, J., Han, X., Jiang, Z., Liu, S., Xu, J., Jiao, P. and Li, Z. (2022) Chromosome-scale assemblies of the male and female Populus euphratica genomes reveal the molecular basis of sex determination and sexual dimorphism. *Commun Biol* **5**, 1186.

Supplemental Table 4. Comparison of statistics of the published T2T genome assemblies in *Populus*.

Species	*P. ussuriensis*	*P. alba* × *P. tremula* var. *glandulosa* clone 84K	*P.* × *deltoids* Danhong	*P. deltoides* NL2-2	*P. lasiocarpa*	*P. nigra* NL-1976	*P. trichocarpa*	ZH1-T2T
**Assembly size (Mb)**	412.13	400.19/416.76	419.4	403.55	436.70/445.01	385.18/390.48	391.76/397.43	506.85/501.23
**Contig N50 (Mb)**	19.5	21.76/23.41	22	22.73	23.30/24.47	22.31/22.05	21.8/20.9	24.99/24.72
**Gap number**	7	2/0	1	0	0/0	0/0	6/2	0/0
**Telomere number**	38	38/38	35	36	36/36	36/36	37/34	38/38
**BUSCO (%)**	98.7	96.67/96.67	98.7	98.8	99.26/99.32	98.7/98.8	98.5/98.2	98.9/98.9
**TEs (%)**	43.18	49.7/50.8	45.22	44.71	51.98/52.44	46.35/47.35	36.02/37.30	58.37/58
**Gene number**	34,953	33,170/33,166	35,232	34,894	36,155/37,012	49,077/50,129	30,089/30,331	38,257/38,176
**Assembly assessment**	—	—	—	QV: 50.30	QV: 58.71/61.51 LAI: 14.29/14.96	QV: 41.57/40.70	LAI: 18.48/18.72	QV: 72.33/73.41 LAI: 20.60/18.72
**Reference**	Liu et al., 2024	Shi et al., 2024	Zhou et al., 2025	Bi et al., 2025	Shen et al., 2025	Liu et al., 2025	Gao et al., 2025	

References

Bi, C., Sun, N., Hou, Z., Dai, X., Wu, H., Han, F., Wang, Z. and Yin, T. (2025) A gap-free reference genome of *Populus deltoides* provides insights into karyotype evolution of Salicaceae. *BMC Biol* **23**, 201.

Gao, W., Wang, S., Jiang, T., Hu, H., Gao, R., Zhou, M. and Wang, G. (2025) Chromosome-scale and haplotype-resolved genome assembly of *Populus trichocarpa*. *Hortic Res* **12**, uhaf012.

Liu, F., Liu, C., Broeck, A.V., Stochlova, P., Jiang, X., Gao, C., Zhang, X., Liu, N. and Huang, Q. (2025) Haplotype-resolved T2T genome assembly of the *Populus nigra* NL-1976. *Sci Data*.

Liu, W., Liu, C., Chen, S., Wang, M., Wang, X., Yu, Y., Sederoff, R.R., Wei, H., You, X., Qu, G. and Chen, S. (2024) A nearly gapless, highly contiguous reference genome for a doubled haploid line of *Populus ussuriensis*, enabling advanced genomic studies. *For Res* (Fayettev) **4**, e019.

Shen, T., Ning, Y., Wang, Y., Song, Z., Xi, M., Pan, H. and Xu, M. (2025) Haplotype-resolved telomere-to-telomere genome assembly of *Populus lasiocarpa* unveils retrotransposon-driven centromere evolution. *The Plant journal : for cell and molecular biology* **123**, e70504.

Shi, T.L., Jia, K.H., Bao, Y.T., Nie, S., Tian, X.C., Yan, X.M., Chen, Z.Y., Li, Z.C., Zhao, S.W., Ma, H.Y., Zhao, Y., Li, X., Zhang, R.G., Guo, J., Zhao, W., El-Kassaby, Y.A., Muller, N., Van de Peer, Y., Wang, X.R., Street, N.R., Porth, I., An, X. and Mao, J.F. (2024) High-quality genome assembly enables prediction of allele-specific gene expression in hybrid poplar. *Plant Physiol* **195**, 652–670.

Zhou, X., Zhang, L., Zhang, M., Wei, H., Bai, Y., Tian, J. and Hu, J. (2025) Genomic selection for growth and wood properties in multi-generation hybrid populations of Populus deltoides. *Hortic Res* **12**, uhaf165.

Supplemental Table 5. Statistics of repeat elements.

Type	Number	Length	Rate (%)
**DNA**	457,527	110,919,479	11.00
**LINE**	73,038	15,943,163	1.58
**SINE**	3,372	724,871	0.07
**LTR**	480,485	446,634,095	44.31
**LTR/Gypsy**	363,006	398,860,194	39.57
**LTR/Copia**	84,035	42,243,818	4.19
**Unknown**	3,813	948,807	0.09
**Total**	1,164,915	586,562,116	58.19

Supplemental Table 6. Statistics of non-coding RNA annotation.

Type		Copy	Average length (bp)	Total length (bp)	% of genome
miRNA		2,035	112	227,107	0.0225
tRNA		1,301	75	97,478	0.0097
rRNA	Total	16,154	1,104	17,828,175	1.7685
	18S	5,227	1,712	8,946,412	0.8875
	28S	7,813	1,083	8,462,340	0.8395
	5.8S	1,122	166	186,672	0.0185
	5S	1,006	119	119,351	0.0118
snRNA	Total	2,704	112	301,971	0.0300
	CD-box	2,364	108	254,984	0.0253
	HACA-box	148	126	18,599	0.0018
	splicing	192	148	28,388	0.0028

Supplemental Table 7. Statistics of the number of rDNAs.

Chr ID	5S	5.8S	18S	28S	Total rDNA
**chr1A**	2	2	0	1	5
**chr1B**	1	0	0	1	2
**chr2A**	0	0	0	1	1
**chr2B**	0	0	0	1	1
**chr3A**	0	0	0	0	0
**chr3B**	0	0	0	0	0
**chr4A**	1	0	1	0	2
**chr4B**	1	0	1	0	2
**chr5A**	0	0	1	0	1
**chr5B**	0	0	1	0	1
**chr6A**	0	0	0	1	1
**chr6B**	0	0	0	1	1
**chr7A**	1	0	0	0	1
**chr7B**	3	0	1	0	4
**chr8A**	1	0	0	0	1
**chr8B**	1	0	0	0	1
**chr9A**	0	643	2709	4050	7402
**chr9B**	0	475	2492	3741	6708
**chr10A**	0	0	0	4	4
**chr10B**	0	0	1	0	1
**chr11A**	0	1	1	2	4
**chr11B**	0	1	1	2	4
**chr12A**	1	0	0	0	1
**chr12B**	1	0	0	0	1
**chr13A**	0	0	0	0	0
**chr13B**	0	0	0	0	0
**chr14A**	0	0	11	2	13
**chr14B**	0	0	0	0	0
**chr15A**	0	0	3	1	4
**chr15B**	0	0	4	1	5
**chr16A**	0	0	0	2	2
**chr16B**	0	0	0	2	2
**chr17A**	546	0	0	0	546
**chr17B**	446	0	0	0	446
**chr18A**	1	0	0	0	1
**chr18B**	0	0	0	0	0
**chr19A**	0	0	0	0	0
**chr19B**	0	0	0	0	0

Supplemental Table 8. Statistics of mapping rates of 113 RNA-seq samples of *Populus euphratica* from public databases.

database	BioProject	Accession	hapA mapping rate	hapB mapping rate
CNGB	PRJCA006811	CRR336890	89.04%	89.13%
	PRJCA006811	CRR336891	91.06%	90.92%
	PRJCA006811	CRR336892	85.67%	85.66%
	PRJCA006811	CRR336893	92.43%	92.39%
	PRJCA006811	CRR336894	92.43%	92.36%
	PRJCA006811	CRR336895	92.05%	91.98%
	PRJCA006811	CRR336896	92.52%	92.42%
	PRJCA006811	CRR336897	90.49%	90.38%
	PRJCA006811	CRR336898	92.89%	92.64%
	PRJCA006811	CRR336899	92.04%	91.99%
	PRJCA006811	CRR336900	92.41%	92.35%
	PRJCA006811	CRR336901	93.46%	93.32%
	PRJCA006811	CRR336902	93.41%	93.15%
	PRJCA006811	CRR336903	90.21%	90.19%
	PRJCA006811	CRR336904	87.97%	87.99%
	PRJCA006811	CRR336905	93.24%	93.08%
	PRJCA006811	CRR336906	92.46%	92.39%
	PRJCA006811	CRR336907	93.23%	93.07%
	PRJCA006811	CRR336908	93.31%	93.13%
	PRJCA006811	CRR336909	93.14%	93.08%
	PRJCA007522	CRR352890	89.51%	89.11%
	PRJCA007522	CRR352891	88.99%	88.63%
	PRJCA007522	CRR352892	89.56%	89.25%
	PRJCA007522	CRR352893	93.21%	93.10%
	PRJCA007522	CRR352894	92.70%	92.55%
	PRJCA007522	CRR352895	92.86%	92.70%
	PRJCA007522	CRR352896	92.20%	92.17%
	PRJCA007522	CRR352897	93.29%	93.22%
	PRJCA007522	CRR352898	90.58%	90.56%
	PRJCA007522	CRR352899	92.78%	92.69%
	PRJCA007522	CRR352900	91.88%	91.84%
	PRJCA007522	CRR352901	92.96%	92.88%
	PRJCA007522	CRR352902	93.01%	92.76%
	PRJCA007522	CRR352903	92.77%	92.52%
	PRJCA007522	CRR352904	93.07%	92.82%
	PRJCA007522	CRR352905	92.28%	92.16%
	PRJCA007522	CRR352906	93.44%	93.32%
	PRJCA007522	CRR352907	93.61%	93.46%
	PRJCA007522	CRR352908	93.26%	93.13%
	PRJCA007522	CRR352909	90.24%	90.10%
	PRJCA007522	CRR352910	92.54%	92.42%
NCBI	PRJNA215888	SRR955312	92.03%	92.72%
	PRJNA215888	SRR956808	92.87%	92.92%
	PRJNA1313092	SRR35200338	92.36%	92.24%

PRJNA1313092	SRR35200339	91.16%	91.04%
PRJNA1313092	SRR35200340	91.33%	91.21%
PRJNA1313092	SRR35200341	90.40%	90.27%
PRJNA1313092	SRR35200344	91.45%	91.32%
PRJNA1313092	SRR35200345	90.56%	90.43%
PRJNA207974	SRR921507	92.53%	93.21%
PRJNA207974	SRR922436	94.05%	94.36%
PRJNA207974	SRR901769	95.42%	95.58%
PRJNA433297	SRR6702217	96.61%	96.47%
PRJNA433297	SRR6702218	97.16%	96.90%
PRJNA433297	SRR6702219	97.21%	96.95%
PRJNA432866	SRR6679094	97.16%	96.90%
PRJNA432866	SRR6679095	97.21%	96.95%
PRJNA215145	SRR952700	92.26%	92.03%
PRJNA215145	SRR952701	87.97%	87.74%
PRJNA215145	SRR952702	90.29%	90.07%
PRJNA215145	SRR952703	93.48%	93.24%
PRJNA215145	SRR952704	89.91%	89.68%
PRJNA215145	SRR952705	86.72%	86.51%
PRJNA215145	SRR952706	92.67%	92.42%
PRJNA215145	SRR952707	89.47%	89.23%
PRJNA215145	SRR952708	88.06%	87.84%
PRJNA215145	SRR952709	69.70%	69.53%
PRJNA215145	SRR952725	96.13%	95.74%
PRJNA215145	SRR952726	95.97%	95.37%
PRJNA215145	SRR952753	85.35%	85.60%
PRJNA215145	SRR952754	95.41%	95.04%
PRJNA542727	SRR9050916	59.84%	60.02%
PRJNA542727	SRR9050917	59.92%	60.08%
PRJNA542727	SRR9050918	90.38%	90.27%
PRJNA542727	SRR9050919	90.61%	90.49%
PRJNA542727	SRR9050920	90.12%	90.02%
PRJNA542727	SRR9050921	77.19%	77.10%
PRJNA542727	SRR9050922	71.49%	71.40%
PRJNA542727	SRR9050923	90.70%	90.58%
PRJNA542727	SRR9050924	89.80%	90.09%
PRJNA542727	SRR9050925	89.39%	89.66%
PRJNA542727	SRR9050926	89.79%	90.07%
PRJNA542727	SRR9050927	64.58%	64.75%
PRJNA390611	SRR5983970	89.75%	89.62%
PRJNA390611	SRR5983971	89.75%	89.62%
PRJNA390611	SRR5983972	91.44%	91.51%
PRJNA390611	SRR5983973	89.68%	89.58%
PRJNA390611	SRR5983974	90.12%	90.01%
PRJNA390611	SRR5983975	92.24%	92.18%
PRJNA390611	SRR5983976	92.23%	92.11%
PRJNA390611	SRR5983997	92.58%	92.59%
PRJNA390611	SRR5983998	92.79%	92.80%
PRJNA390611	SRR5984029	91.16%	91.08%

PRJNA390611	SRR5984052	89.75%	89.80%
PRJNA390611	SRR5984065	91.94%	92.02%
PRJNA390611	SRR5984066	87.79%	87.77%
PRJNA484685	SRR8741502	88.12%	88.43%
PRJNA484685	SRR8741503	90.25%	90.54%
PRJNA484685	SRR8741504	91.54%	91.65%
PRJNA484685	SRR8741505	89.21%	89.48%
PRJNA484685	SRR8741506	89.87%	90.14%
PRJNA484685	SRR8741510	94.52%	94.70%
PRJNA484685	SRR8741511	95.09%	95.11%
PRJNA484685	SRR8741524	95.52%	95.75%
PRJNA484685	SRR8741525	94.19%	94.42%
PRJNA484685	SRR8741526	93.91%	94.04%
PRJNA484685	SRR8741527	94.16%	94.45%
PRJNA484685	SRR8741528	95.65%	95.67%
PRJNA484685	SRR8741529	89.86%	90.14%
PRJNA484685	SRR8741530	94.00%	94.26%
PRJNA484685	SRR8741508	91.74%	91.69%
PRJNA484685	SRR8741509	89.83%	89.85%
PRJNA484685	SRR8741507	89.89%	89.60%

Supplemental Table 9. Statistics of gene annotation from public databases.

Item	Count	Percentage
**All**	76,433	100.00%
**Annotation**	74,275	97.18%
**KEGG**	30,740	40.22%
**Pathway**	13,557	17.74%
**Nr**	73,254	95.84%
**Uniprot**	72,919	95.40%
**GO**	60,879	79.65%
**KOG**	1,058	1.38%
**Pfam**	60,160	78.71%
**Interpro**	69,205	90.54%

Supplemental Table 10. Comparison of the regeneration efficiency.

Methods	leaf disks	callus induction	shoot induction	root induction
**Indirect**	50	134/50 (268%)	109/134 (81.3%)	109/109 (100%)
**Direct**	50	0	48/50 (96%)	48/48 (100%)

Supplemental Table 11. Summary of the editing types at each target site in the direct and indirect regeneration systems.

Mutation type	Direct regeneration		Indirect regeneration		Phenotype
	T1	T2	T1	T2	
**Biallelic**	2	4	29	28	albinism
**Homozygous**	0	0	19	20	albinism
**Heterozygous**	5	2	1	2	albinism
**Chimeric**	31	28	2	2	pale
**Unmodified**	30	34	12	11	green
**Number of transgenic plants**	68		63		
**Mutation rate**	55.8%	50.0%	81.0%	82.5%	

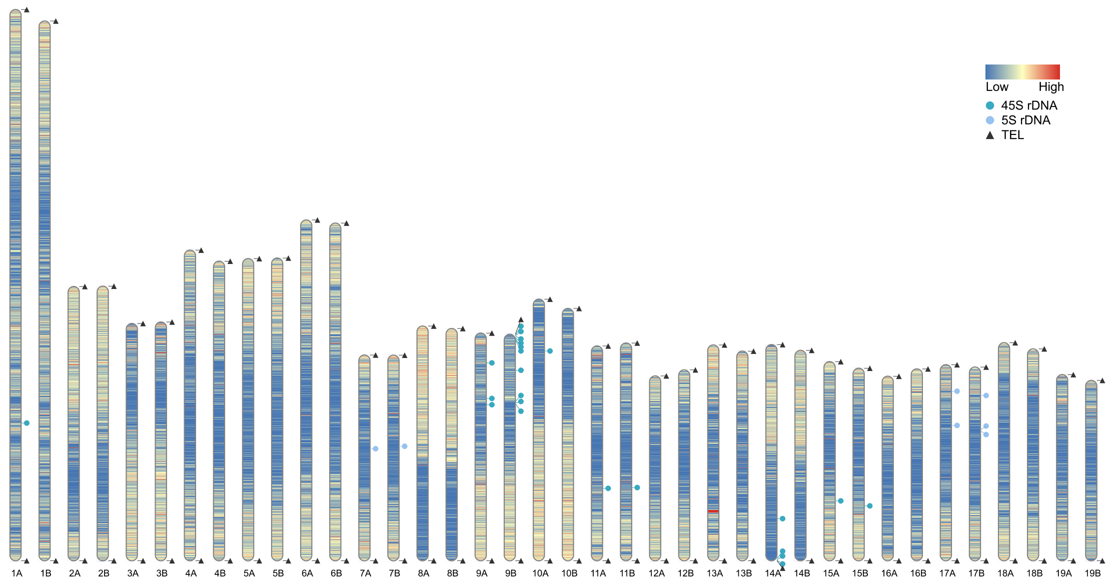

Supplemental Figure 1. 38 chromosomes of *P. euphratica* (2n = 38). The gradient color on the chromosome represents gene density. Blue indicates low gene density and high repetitive sequence density, and red indicates high gene density. Green circles indicate 45S rDNA, and blue circles represent 5S rDNA. Triangles represent the telomeres.

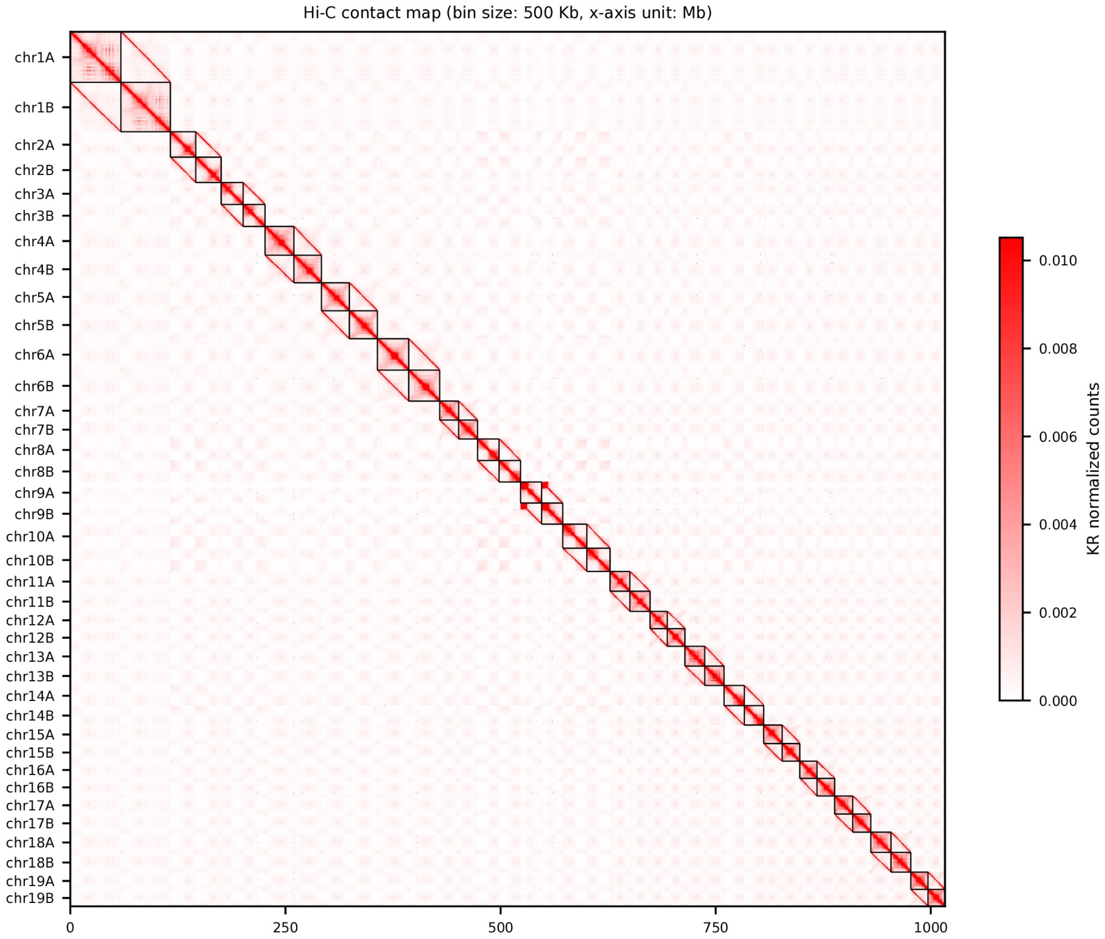

Supplemental Figure 2. Interaction frequency distributions of Hi-C linkage groups. The KR normalized counts of Hi-C data between any pair of 500-kb non-overlapping bins were calculated.

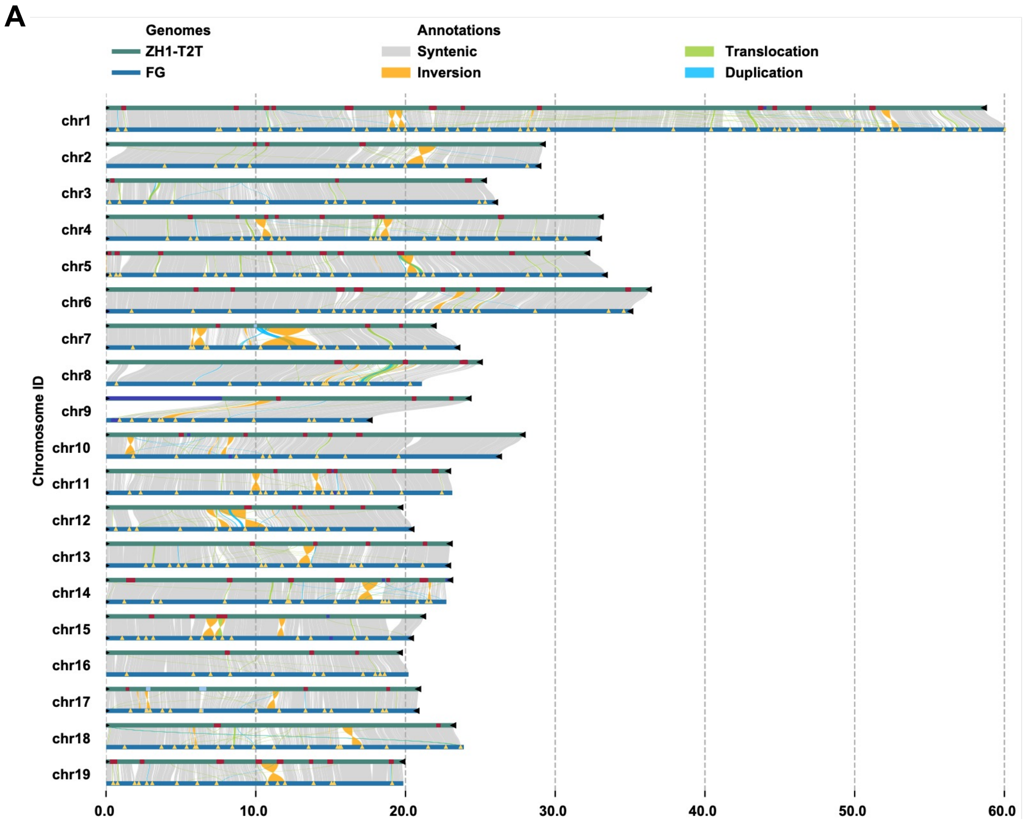

B

ID	Gap number	Gene number in gaps
chr1	45	98
chr2	13	14
chr3	13	22
chr4	24	40
chr5	23	77
chr6	23	74
chr7	14	13
chr8	14	23
chr9	13	8
chr10	9	35
chr11	17	27
chr12	14	18
chr13	18	12
chr14	17	94
chr15	14	36
chr16	11	9
chr17	14	5
chr18	19	19
chr19	16	59
Total	331	683

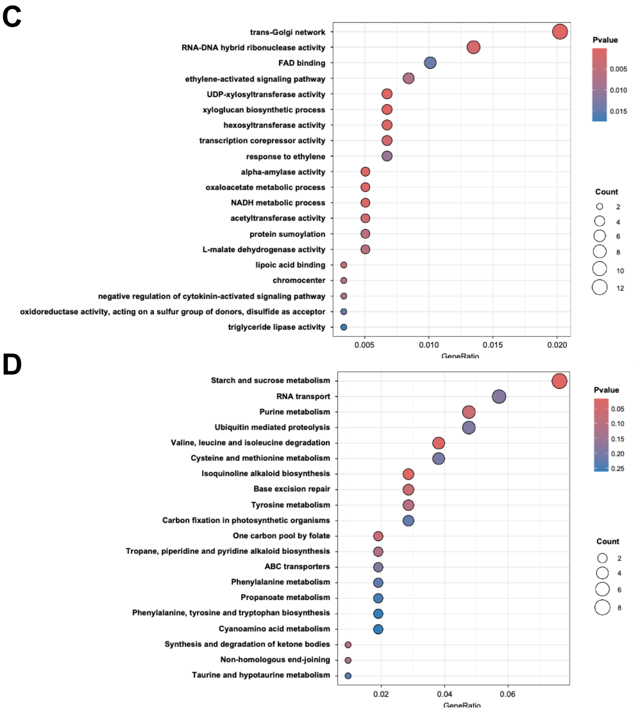

Supplemental Figure 3. Comparison of *P. euphratica* genomes. (A) Genome collinearity of ZH1-T2T and female genome (FG). Black triangles indicate telomeres, yellow triangles represent gap sequences, light blue denotes 5S rDNA, dark blue denotes 45S rDNA, and red indicates gap-associated genes. (B) Summary of gap-associated genes. (C) GO enrichment of gap-associated genes. (D) KEGG enrichment of gap-associated genes.

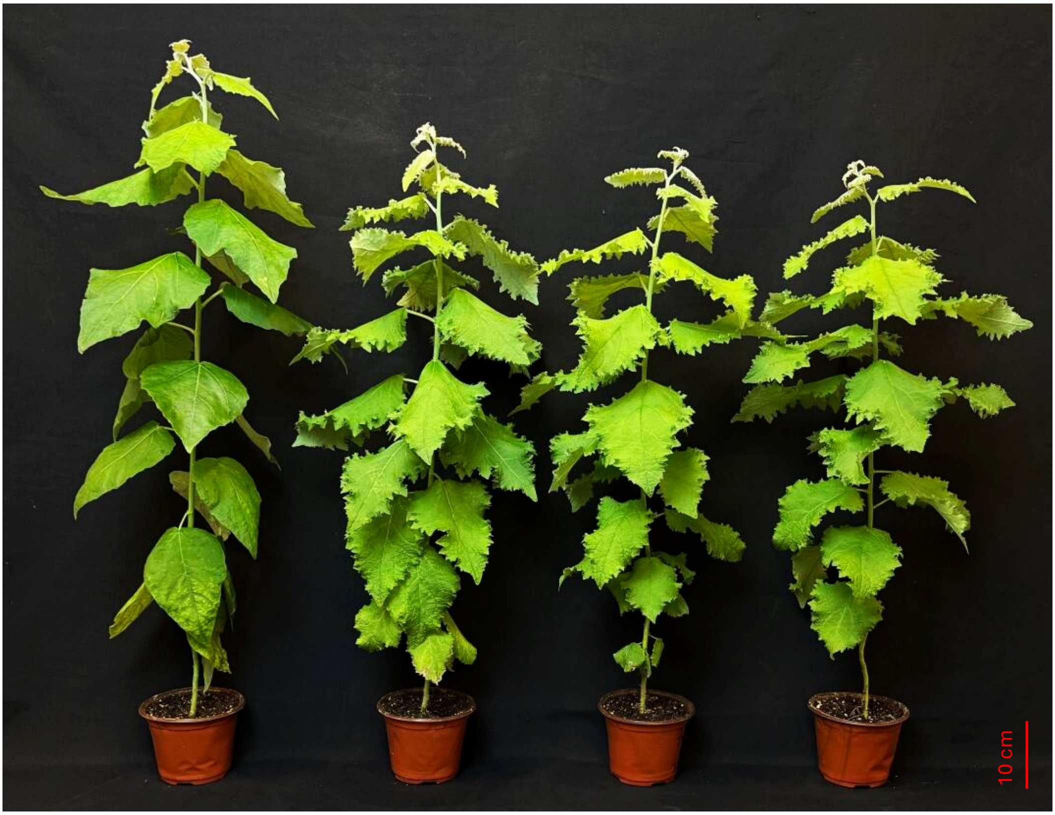

CK *PagmiR319-OE#1* *PagmiR319-OE#2* *PagmiR319-OE#3*

Supplemental Figure 4. Morphology of *PagmiR319*-overexpressing 84K poplar.

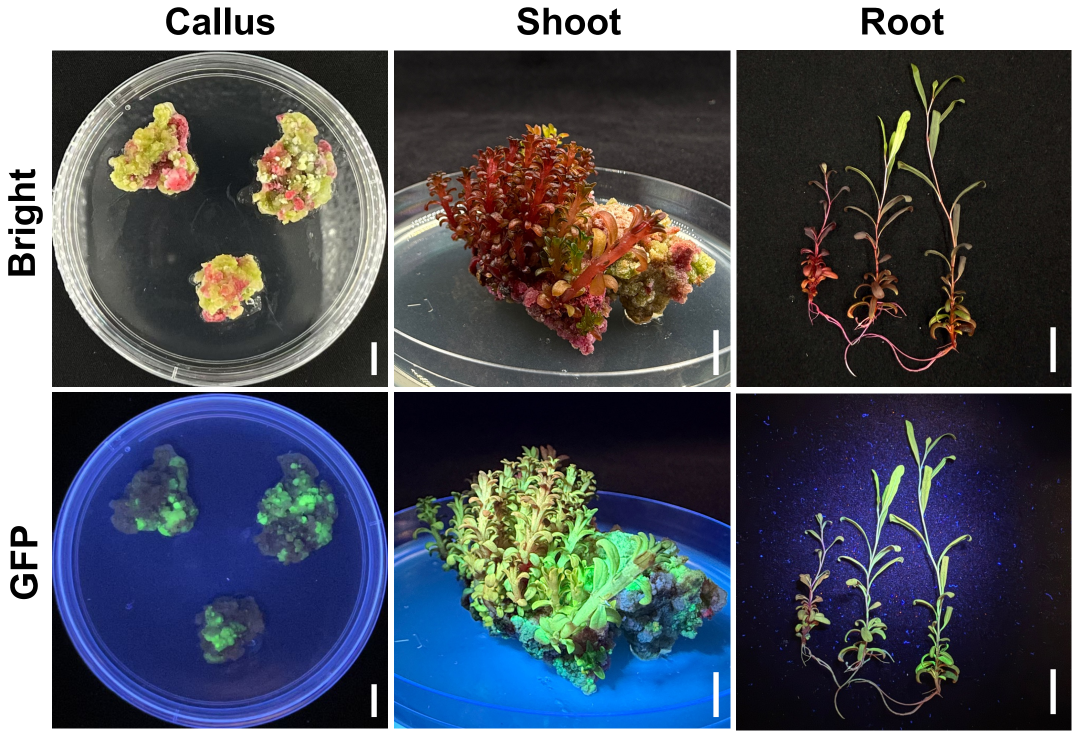

Supplemental Figure 5. Overexpression of RUBY in *P. euphratica.* The RUBY red is present in the callus, shoot, and root. Bar = 1 cm.

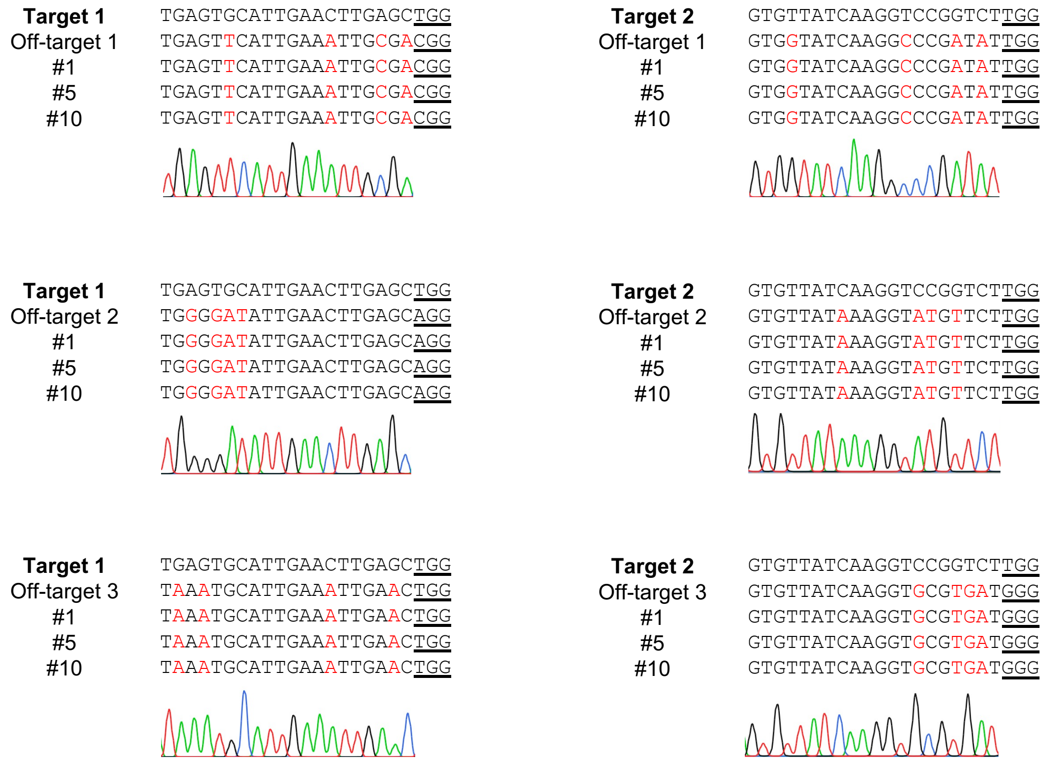

Supplemental Figure 6. Sequencing analysis of potential off-target sites of two *PePDS* sgRNAs.

Supplemental Note 1: Genome sequencing, assembly, and annotation

Genome sequencing

For PacBio HiFi sequencing, high-molecular-weight genomic DNA (gDNA) from *Populus euphratica* clone "ZH1" was prepared from leaves using the CTAB method and purified with the Grandomics Genomic kit, following the manufacturer's standard operating procedure. SMRTbell target size libraries were constructed for sequencing according to PacBio's standard protocol (Pacific Biosciences, CA, USA) using 15kb preparation solutions. Sequencing was performed on a PacBio Revio instrument using Sequencing Primer V5 and Revio Binding Kit 2.2 in Grandomics (Wuhan, China). Raw PacBio HiFi subreads were filtered using the software ccs (v6.0.0; parameters: –min-passes 3 –min-snr 2.5 –top-passes 60; https://github.com/PacificBiosciences/ccs). Subreads with fewer than 3 passes and an SNR below 2.5 were removed, yielding high-quality CCS reads for subsequent analysis. For the Nanopore long-read sequencing, gDNA samples were extracted using a Genomic DNA extraction kit (Catalog No. 13323, QIAGEN) and selected (> 100 kb) with the SageHLS HMW library system (Sage Science) and then processed using the Ligation sequencing 1D Kit (Catalog No. SQK-LSK109, Oxford Nanopore Technologies, Oxford, UK) according to the manufacturer's instructions. DNA libraries were constructed and sequenced on the PromethION (Oxford Nanopore Technologies). Filtering of raw data was performed by removing failed reads (sequences with an average quality value less than 7). Subsequently, Filtlong (v0.2.4; https://github.com/rrwick/Filtlong) was utilized to filter out fragments shorter than 10 kb, yielding effective data (pass reads) for further analysis. Porechop (v0.2.4; https://github.com/rrwick/Porechop) was then employed to filter adapter sequences, followed by another round of filtering using Filtlong (v0.2.4; https://github.com/rrwick/Filtlong), retaining reads with lengths ≥ 30 kb and mean read quality scores > 90% for assembly. For the Hi-C sequencing, the sample pretreatment and library preparation were performed according to the standard protocol. The qualified libraries were sequenced on the DNBSEQ-T7RS platform. Raw reads were filtered using fastp (v0.21.0) to remove adapter sequences and low-quality reads, thereby obtaining high-quality clean data. Clean data were aligned to the reference genome and filtered using the software HICUP (v0.8.0; http://www.bioinformatics.babraham.ac.uk/projects/hicup/). The gDNA was extracted using the CTAB method for short-read sequencing. Sequencing libraries were constructed using the MGIEasy Universal DNA Library Prep Kit V1.0 (Catalog No.

1000005250, MGI) according to the standard protocol. The qualified libraries were sequenced on the DNBSEQ-T7RS platform. Raw reads were filtered using fastp (v0.21.0) to discard low-quality reads.

Genome assembly

ONT ultra-long, PacBio HiFi, and Hi-C data were used for initial assembly with Hifiasm (version 0.25.0-r726; https://github.com/chhylp123/hifiasm). HapHiC (v1.0.7) was employed for Hi-C-assisted scaffolding. Subsequently, Juicebox (v1.11.08) was used for manual ordering and orienting of the sequenced and oriented contigs to generate the reviewed assembly. For the centromeric region, error correction was performed using ONT data with the k-mer anchoring method. ONT reads (≥ 40 kb) were aligned to pseudochromosomes using minimap2 (v2.28-r1209) to obtain bam files. To avoid over-correction of centromeric sequences, 21-kmers that appeared once in the assembly and 14 to 46 times in the short reads were identified, and the bam file was marked using the subprogram filter_by_marker_nosplit.sh from the software T2T-Polish (https://github.com/malonge/T2T-Polish). Error correction was carried out using medaka consensus (v1.5.0; parameters: –model r941promhacg507 –batch_size 200; https://github.com/nanoporetech/medaka). The stitched consensus sequence was obtained using medaka stitch (v1.5.0; https://github.com/nanoporetech/medaka). The above steps were repeated twice. Further error correction was performed using the short-read data with the software deepVariant (v1.3.0). The short-read data were aligned to the previously corrected genome to produce BAM files using bwa mem (v0.7.17-r1188) and samtools (v1.9). A VCF file was generated using deepvariant (v1.3.0; parameters: –model_type=WGS) and filtered using bcftools (v1.15; parameters: view -e ‘type="ref"’, view -i ‘QUAL>1 && (GT="AA" || GT="Aa")’), and normalized with bcftools norm to left-align and standardize the VCF file. The VCF file was then compressed and indexed. Consensus sequences were exported using bcftools consensus function. For the telomere correction, Winnowmap (v1.11, parameters: k=15, –MD) (Chirag et al. 2020) was used to align all ONT reads to the reference genome, collecting all reads that aligned once within the last 50 bp of each chromosome. The occurrence frequency of the telomere repeat motifs (CCCTAAA’/‘TTTAGGG) in each read was calculated. The read with the highest frequency was designated the reference, with the others defined as queries. The software medakaconsensus (v1.2.1, parameters: -m r941*min*high_g360; https://github.com/nanoporetech/medaka) was then employed to reassemble the reference telomere reads and query telomere reads to obtain consensus

sequences. Using nucmer (v3.1), these consensus sequences were separately aligned to each chromosome, and the best alignments were used to replace the terminal telomeric sequences if the identity exceeded 80% and the alignment region was within the last 20 kb of each chromosome. For gap filling, the software Winnowmap (v1.11, parameters: k=15, -MD) was used by integrating the Hifiasm assembly, ONT, and HiFi data to close gap regions. Finally, Winnowmap2 was used to map HIFI reads (≥10 kb) to the gap-filled genome with parameters: k=15, greater-than distinct=0.9998, -MD, -ax map-pb. Filtered the alignment results using samtools view (v1.10, parameter: -F 256). Removed chimeric alignment segments using falconc bam-filter-clipped -t -F 0x104. Performed error correction using a specialized branch of racon (v1.6.0, -L -u, https://github.com/isovic/racon/tree/liftover) based on the filtered alignment information. For genome assessment, the short-read and long-read data were aligned to the assembled genome using bwa mem (v0.7.18) and minimap2 (v2.28), respectively. The alignment rate and coverage were then calculated.

Genome annotation

For the annotation of repeat sequences, RepeatModeler (v2.0.4) was first used to *de novo* predict model sequences based on the genome sequence itself. Simultaneously, LTRFINDER (v1.07) and LTRharvest (v1.62) were utilized to predict LTR sequences. LTRretriever (v2.9.0) was then applied to remove redundancies from the predicted sequences obtained by both methods, resulting in non-redundant LTR sequences. The two datasets were combined to form a *de novo* repeat sequence library. Subsequently, TEClass (v2.1.3) was used to redefine the Unknown content within this library, after which the RepBase database (v20181026) and the *de novo* library were merged and analyzed using RepeatMasker (v4.1.5) for comparison and prediction of repeat sequences, yielding the merged *de novo* and RepBase results; further, RepeatProteinMask in RepeatMasker (v4.1.5) was employed to predict TE_protein type repeat sequences, generating TE proteins results; finally, all repeat prediction results were combined and deduplicated to obtain the final genomic dispersed repeat sequence set. Tandem repeat prediction was performed using TRF (v4.09) and MISA (v2.1). tRNA sequences in the genome were predicted using tRNAscan-SE (v1.23). rRNA prediction was performed using an rRNA database. Non-coding RNA (ncRNA) sequences in the genome were identified using INFERNAL (v1.1.2) based on the Rfam database.

This study employed a combined approach to gene structure prediction, integrating

transcriptomic, homology, and de novo predictions. Transcriptome prediction used both full-length and short-read transcriptomic data. The transcriptome data were aligned to the genome using hisat2 (v2.1.0). Subsequently, stringtie (v2.1.4) was used to reconstruct the transcripts obtained, and TransDecoder (v5.1.0) predicted coding frames from the predicted transcript regions, ultimately yielding predicted coding genes. Homology prediction involved selecting protein sequence files from multiple closely related species for predictive analysis. tblastn (v2.7.1) was used to align homologous protein sequences to the genome, followed by Exonerate (v2.4.0), which predicted transcripts and coding regions based on alignment results. The *de novo* prediction was conducted on repeat-masked genomes using Augustus (v3.3.2) and GlimmerHMM (v3.0.4). MAKER (v2.31.10) integrated gene sets predicted by various methods into a final dataset. Gene function annotation employed the following two approaches. (1) Sequence similarity search. Protein sequences were blasted against existing protein databases (Uniprot and NR) and the metabolic pathway database KEGG using diamond blastp (v2.0.11.149), to obtain functional information and potential metabolic pathways associated with the proteins. Functional annotations were further assigned to KEGG ORTHOLOGY and PATHWAY using KOBAS (v3.0). Additionally, Gene Ontology annotations were derived from the correspondence between proteins in the Uniprot database and Gene Ontology terms. (2) Motif similarity search. InterProScan (v5.55-88.0, default parameters) was used to perform blast against sub-databases of InterPro, including CDD, Gene3D, Hamap, Panther, Pfam, Phobius, Pirsf, Pirsr, Prints, Prosite, Sfld, Smart, Superfamily, Tigrfam, and Tmhmm, to identify conserved sequence motifs and structural domains within proteins. Furthermore, hmmscan (v3.3.2; parameters: –cpu 16 -E 1e-5) was used to predict conserved sequences, motifs, and structural domains in proteins.

Supplemental Note 2: Plant regeneration and transformation

Plant pretreatment

The plant material used in this study was *Populus euphratica* Oliv. clone "ZH1", cultivated at the Pingshan experimental base of Zhejiang A&F University in Hangzhou, China. Healthy leaves were selected and surface-cleaned with running water for 2 hours. Under a sterile workbench, the leaves were treated with 75% ethanol for 10 seconds, rinsed three times with sterile water, and then washed with 0.2% mercuric chloride solution for 10 minutes. Subsequently, the leaves were rinsed 5 times with sterile water, dried on sterile filter paper, and cut into small pieces, approximately 0.5 cm in size, for later use.

Plant regeneration

Leaf pieces were inoculated onto the callus induction medium and cultured in the dark for approximately 2 weeks, resulting in callus tissue about 0.5 cm in size. Well-developed, loosely textured callus tissue was transferred to the shoot induction medium and then exposed to light to induce differentiation. After about 2–3 weeks, adventitious buds began to emerge. These elongated adventitious buds were subsequently transferred to the rooting medium for root formation. In addition to the indirect organogenesis pathway described above, explants could be inoculated onto the shoot induction medium to directly differentiate adventitious buds at the wound site. This direct pathway exhibited higher regeneration efficiency and shorter regeneration cycles, with adventitious buds typically differentiating within about a month. The growth room was set to 16 h of white light at 150 μmol m^{-2} s^{-1} irradiance, followed by 8 h of darkness at 20°C.

Plant transformation

(1) Young leaves of the sterile seedlings were cut into small pieces, approximately 0.5 cm in length, and, with or without a 2-day pre-culture on co-culture induction medium (CCIM).

(2) *Agrobacterium* cells (GV3101) carrying a target vector were grown overnight at 25°C in liquid LB medium supplemented with appropriate antibiotics.

(3) The *Agrobacterium* cells were collected by centrifugation at 3000 rpm for 10 min and resuspended in induction medium (IM) to an OD600 of 0.3–0.4, then induced by shaking (50–100 rpm) for 30 min at room temperature.

(4) Leaf pieces were soaked for 10 min at room temperature in the bacterial suspension containing the target vector, with gentle shaking.

(5) After removal, the leaf pieces were blotted dry with sterile filter paper and placed on CCIM free of selection pressure, incubated at 25°C in the dark for 2 days.
(6) Leaf pieces were washed several times with double-distilled water.

For the indirect regeneration process:

(7) Leaf pieces were transferred to the callus-induction media (CIM) with selection pressure for continued dark cultivation, during which calli approximately 0.5 cm in diameter formed within approximately 2–3 weeks.
(8) These calli were then subcultured onto the shoot-induction media (SIM) under selection pressure. During this period, the calli would turn green and become harder.
(9) After 2–4 weeks, the approximately 1 cm-long shoots in good growth were excised and transferred to the root-induction media (RIM), where roots would develop within about 7–10 days.

For the direct regeneration process:

(7) Shoot regeneration was induced on SIM with selection pressure for 2–3 weeks.
(8) Then, the approximately 1 cm-long shoots in good growth were excised and transferred to the RIM with selection pressure. Root induction takes about 7–10 days.

Positive identification

The positive transgenic plants could be selected by eYGFPuv fluorescence. Genomic DNA from the knockout plants was extracted and used to evaluate the editing conditions by Sanger DNA sequencing.

Media

CCIM: WPM + 0.5 g/L MES + 0.65 g/L calcium gluconate + 0.1 g/L inositol + 100 μM AS + 20 g/L sucrose +7 g/L Agar; pH 5.9

IM: WPM + 0.5 g/L MES + 0.65 g/L calcium gluconate + 0.1 g/L inositol + 100 μM AS + 1.0 mg/L 2,4-D + 0.10 mg/L KT + 20 g/L sucrose; pH 5.6

CIM: WPM + 0.5 g/L MES + 0.65 g/L calcium gluconate + 0.1 g/L inositol + 1.0 mg/L 2,4-D + 0.12 mg/L KT + 20 g/L sucrose +7 g/L Agar (300 mg/L Tim, 5 mg/L G418 or 50 mg/L Kana); pH 5.9

SIM: WPM + 0.5 g/L MES + 0.65 g/L calcium gluconate + 0.1 g/L inositol + 0.5 mg/L 6-BA + 0.02 mg/L TDZ + 20 g/L sucrose + 7 g/L Agar (300 mg/L Tim, 5 mg/L G418 or 50 mg/L Kana); pH 5.9

RIM: WPM + 0.14 mg/L NAA + 0.2 g/L AC + 10 g/L sucrose +7 g/L Agar (300 mg/L Tim, 5 mg/L G418 or 50 mg/L Kana); pH 5.9

Supplemental Note 3: Plasmid design and construction

Plasmid design

To efficiently overexpress one or more genes in plants, we designed a multi-gene expression vector, pXHKFG, including a kanamycin resistance gene (K), a FLAG tag (F), and an *eYGFPuv* gene (G). First, a 3×FLAG tag sequence flanked by EcoRI and MluI restriction sites was synthetically constructed and inserted into the pK2GW7 vector (Karimi et al., 2002) using recombination technology. Subsequently, the vector was linearized using AatII and PmeI. An eYGFPuv cassette sequence (Chin et al., 2018) was synthetically generated and then integrated into the linearized vector via homologous recombination to yield the pXHKFG overexpression vector.

To efficiently edit one or more genes in plants, we designed a multi-target CRISPR/Cas9 system, pXHGCK, including an *eYGFPuv* gene (G), a Cas9 cassette (C), and a kanamycin resistance gene (K). First, the pCAS9-TPC vector (Fauser et al., 2014) was linearized using HindIII. The kanamycin resistance cassette sequence was cloned from the pPZP111 vector (Hajdukiewicz et al., 1994) and subsequently inserted into the linearized vector via homologous recombination. Then, the vector was linearized with EcoRI, and the eYGFPuv cassette was inserted via homologous recombination into the linearized vector to generate the pXHGCK gene-editing vector.

To broaden field application, kanamycin resistance can be replaced with herbicide resistance (*bar*), yielding the pXHBFG overexpression vector and the pXHGCB gene-editing vector.

Plasmid construction

For miR319 overexpression vector construction, the miR319 precursor was cloned from *Populus euphratica* and inserted into pXHKFG by EcoRI linearization and homologous recombination to achieve the pXHKFG-PemiR319 overexpression vector.

For RUBY expression vector construction, the first RUBY-related gene *CYP76AD1* was cloned and inserted into pXHKFG by EcoRI linearization and homologous recombination to produce the pXHKFG-CYP76AD1 vector. The other two RUBY-related genes *DODA* and *DOPA5GT* were cloned and inserted into pEn-P2A entry vector, respectively, by BbsI linearization and homologous recombination to fuse the P2A cleavage peptide to the N-terminus of the target gene (Kim et al., 2011). Then the P2A-DODA and P2A-DOPA5GT cassettes were cloned and inserted into the pXHKFG-CYP76AD1 vector by EcoRI and MluI linearization and multiplex homologous recombination to produce the pXHKFG-RUBY overexpression vector.

For the construction of the *PDS* gene-editing vector, two target sites in the *PDS* gene were selected from the *P. euphratica* genome using CRISPR-Local (Sun et al., 2019). The guide RNA sequences of the target sites were synthesized and inserted into pEn-C1.1 entry vector (Fauser et al., 2014). Then two gRNA cassettes were cloned from entry vector and inserted into pXHGCK by SmaI and XbaI linearization and multiplex homologous recombination to produce the pXHGCK-PePDS gene-editing vector.

References

Chin, D.P., Shiratori, I., Shimizu, A., Kato, K., Mii, M., and Waga, I. (2018). Generation of brilliant green fluorescent petunia plants by using a new and potent fluorescent protein transgene. *Scientific reports* **8**:16556.

Fauser, F., Schiml, S., and Puchta, H. (2014). Both CRISPR/Cas-based nucleases and nickases can be used efficiently for genome engineering in Arabidopsis thaliana. *The Plant journal : for cell and molecular biology* **79**:348–359.

Hajdukiewicz, P., Svab, Z., and Maliga, P. (1994). The small, versatile pPZP family of Agrobacterium binary vectors for plant transformation. *Plant molecular biology* **25**:989–994.

Karimi, M., Inze, D., and Depicker, A. (2002). GATEWAY vectors for Agrobacterium-mediated plant transformation. *Trends in plant science* **7**:193–195.

Kim, J.H., Lee, S.R., Li, L.H., Park, H.J., Park, J.H., Lee, K.Y., Kim, M.K., Shin, B.A. and Choi, S.Y. (2011) High cleavage efficiency of a 2A peptide derived from porcine teschovirus-1 in human cell lines, zebrafish and mice. *PloS one* **6**, e18556.

Sun, J., Liu, H., Liu, J., Cheng, S., Peng, Y., Zhang, Q., Yan, J., Liu, H.J. and Chen, L.L. (2019) CRISPR-Local: a local single-guide RNA (sgRNA) design tool for non-reference plant genomes. *Bioinformatics* **35**, 2501–2503.
